# Aqueous copper geochemistry shapes the sediment microbial resistome in a recovering stream

**DOI:** 10.1111/1758-2229.70045

**Published:** 2024-11-27

**Authors:** Paul G Helfrich, Johnathan Feldman, Eva Andrade‐Barahona, Isaiah Robertson, Jordan Foster, Renee Hofacker, Gavin Dahlquist Selking, Cody S Sheik, Alysia Cox

**Affiliations:** ^1^ Laboratory Exploring Geobiochemical Engineering and Natural Dynamics (LEGEND), Department of Chemistry & Geochemistry Montana Technological University Butte Montana USA; ^2^ Earth Science and Engineering Ph.D. Program Montana Tech Butte Montana USA; ^3^ Department of Soil and Crop Science Texas A&M University College Station Texas USA; ^4^ Biology Department and Large Lakes Observatory University of Minnesota‐Duluth Duluth Minnesota USA

## Abstract

Aqueous metals are pervasive contaminants associated with historical mining. We produced and examined 16 metagenomes from a contaminated creek to investigate how anthropogenic metal contamination shapes the functional profiles of microbial communities. We then incorporated the metagenomic profiles and concurrently collected geochemical context into a multivariate model to examine correlations between stream geochemistry and microbial functional potential. Integrating the metagenomes with full geochemical profiles emphasised that even low metalloid concentrations shaped microbial functionality, seasonal shifts in copper bioavailability and arsenic exposure correlated with genetic variation, and copper resistomes were spatiotemporally distinct. This study provides new insights into microbial metabolic potential and microbe‐metal(loid) interactions.

## Graphical abstract

We examined genes related to microbial survival under copper stress in a mining‐contaminated creek. Our model suggests that microbial copper resistance is related to both copper and arsenic exposure.

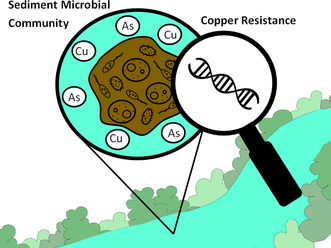

## INTRODUCTION

Microbes are the foundation of all aquatic ecosystems (Grossart et al., 2020). Microbial metabolism drives the biogeochemical flow of carbon, nitrogen, oxygen, sulfur and other elements (Hernandez et al., 2021). Microbial communities readily adapt to changing conditions and develop novel resistance mechanisms that reflect environmental shifts (Guan et al., 2017). Microbial communities play a key role in restoration ecology because they underpin the ecosystem services such as clean water and higher trophic functionality that restoration interventions target (Cavicchioli et al., 2019; Haines et al., 2002; Kallmeyer et al., 2012; Singh Rawat et al., 2023). Moreover, many microbial processes are poorly understood and underrepresented in policy development (Cavicchioli et al., 2019). Further research assessing microbial ecology is essential for future ecological management (Gu, 2021; Hutchins et al., 2021; Treseder et al., 2012; Wang & Gu, 2021).

Model development linking environmental stressors to biological responses is rapidly advancing (Van Genderen et al., 2015). Environmental regulation agencies are increasingly integrating ecotoxicological assessments based on sentinel macroinvertebrates, fish, and algae into standard management practices (Besser et al., 2015; Farley et al., 2015; Meyer et al., 2015). Comparable microbial toxicological frameworks are comparatively underdeveloped (Treseder et al., 2012). If microbiomes are to be widely utilised in environmental management, innovation in biogeochemistry is necessary to clarify microbial responses to stress (Cavicchioli et al., 2019).

Recent advances in omics technologies, genomics, transcriptomics, proteomics, and metabolomics, offer significant potential in improving microbial ecotoxicology (Biales et al., 2015). Methodological frameworks that integrate geochemistry and omics microbiology could be useful in understanding how anthropogenic activity alters aquatic ecology. Pairing comprehensive geochemical measurements and shotgun metagenomic sequencing is an attractive methodology for linking geochemical changes to ecological responses (Li, Hu, et al., 2021; Nishiyama et al., 2018). Geochemical speciation modelling predicts chemical speciation and bioavailability (Arteaga‐Pozo, 2022; Feldman, 2019). Shotgun metagenomic sequencing provides a comprehensive insight into putative microbial functionality (Zhang et al., 2021). Functional profiles reveal gene assemblages that microbial communities use to resist geochemical stressors, resistomes (Jiang et al., 2021; Li, Chen, et al., 2021; Salam, 2020; Salam et al., 2020). Geochemically informed microbial statistical models streamline biogeochemical exploration (Weeks et al., 2023).

Anthropogenically impacted areas are ideal for developing and testing new ecotoxicological frameworks (Schmitt‐Jansen et al., 2008). One of North America's most polluted watersheds is the Clark Fork River in southwest Montana (Axtmann & Luoma, 1991). The river begins as Blacktail Creek before merging with several smaller tributaries to form Silver Bow Creek near Butte, MT. Extensive copper and molybdenum mining polluted the watershed in the 1900s (Benner et al., 1995; Gammons et al., 2005). Contamination devastated the area's terrestrial and aquatic ecosystems, largely expatriating aquatic invertebrates and salmonids from the creek (Chadwick et al., 1986). Remediation efforts removed metal‐rich benthic riverine and riparian sediments during the 1980s (Mason et al., 2012). Metal contamination still impacts the creek (Gammons et al., 2005). Some dissolved metal(loid) levels (copper, zinc and arsenic) rise during storms and when contaminated groundwater enters the stream (Gammons et al., 2005; Lund, 2018; Radar, 2019; Robertson, 2019).

Although stream physicochemical conditions have improved, ecological recovery is ongoing (Moore & Langner, 2012; Moore & Luoma, 1990). Macroinvertebrate community analysis suggests that organic enrichment limits the benthic biota (Stagliano, 2020). Increasing temperatures, low dissolved oxygen (DO) levels, and altered water chemistry constrain trout population recovery (Saffel et al., 2018). Very little is known about the local microbiota. Incubation experiments suggest that local microbial communities exhibit unusual, spatially distinct metabolisms (Robertson, 2019). Interactions between the area's microbial communities and metal contamination are uncharacterised. The ecosystem's evolving state suggests that although remediation efforts altered the drainage, the underlying ecology may still be impacted. Surveying the creek's microbiota could be useful for understanding how water quality shapes the foundational microbial communities and provide relevant insight into microbial ecotoxicology in other mining‐impacted areas.

We investigated microbe‐copper interactions by integrating comprehensive geochemical surveys with metagenomic sampling. We focused on microbial metabolic potential related to one of the area's primary contaminants, copper (the copper resistome). We (1) modelled copper speciation across a temporospatial gradient, (2) developed a geochemical‐metagenomic model to examine geochemical controls on copper resistance, and (3) investigated potential copper‐arsenic stress on microbial communities. This multidisciplinary approach presents new insights into changing copper speciation, metal(loid) interactions, and a comprehensive framework for linking biogeochemistry to metabolic potential.

## EXPERIMENTAL PROCEDURES

### Sampling materials preparation

Sample collection materials were trace metal (TM) cleaned and prepared as previously described (Dahlquist Selking et al., 2023). Briefly, sampling equipment was washed using successive treatments of 1% (v/v) citranox/water, 10% v/v HCl/water (Baker Analyzed™ A.C.S. Reagent), pH 2 HCl and either deionised tap water(DI) or 18.2 MΩ‐cm water (ultra‐pure water). All cleaning and blank procedures were conducted using DI water before February 2016, after which ultra‐pure water was used. Amber borosilicate glass vials and open‐top septa caps used for dissolved inorganic (DIC) and organic (DOC) carbon analysis were rinsed with DI water seven times, soaked in 10% v/v HCl/water (Baker 262 Analyzed™ A.C.S. Reagent) for 72 h and again rinsed 7 times with DI water. The vials were then wrapped in aluminium foil and placed in a muffle furnace at 450°C for 4 h. The caps were placed on muffled tin foil and dried in the high‐efficiency particulate air (HEPA) 100 filter hood. Muffled vials were then immediately capped in the HEPA hood. Vials designated for DOC samples were spiked with 85 μL of concentrated phosphoric acid (Baker Analyzed™ A.C.S. Reagent).

### In situ physiochemical characterisation

**FIGURE 1:**
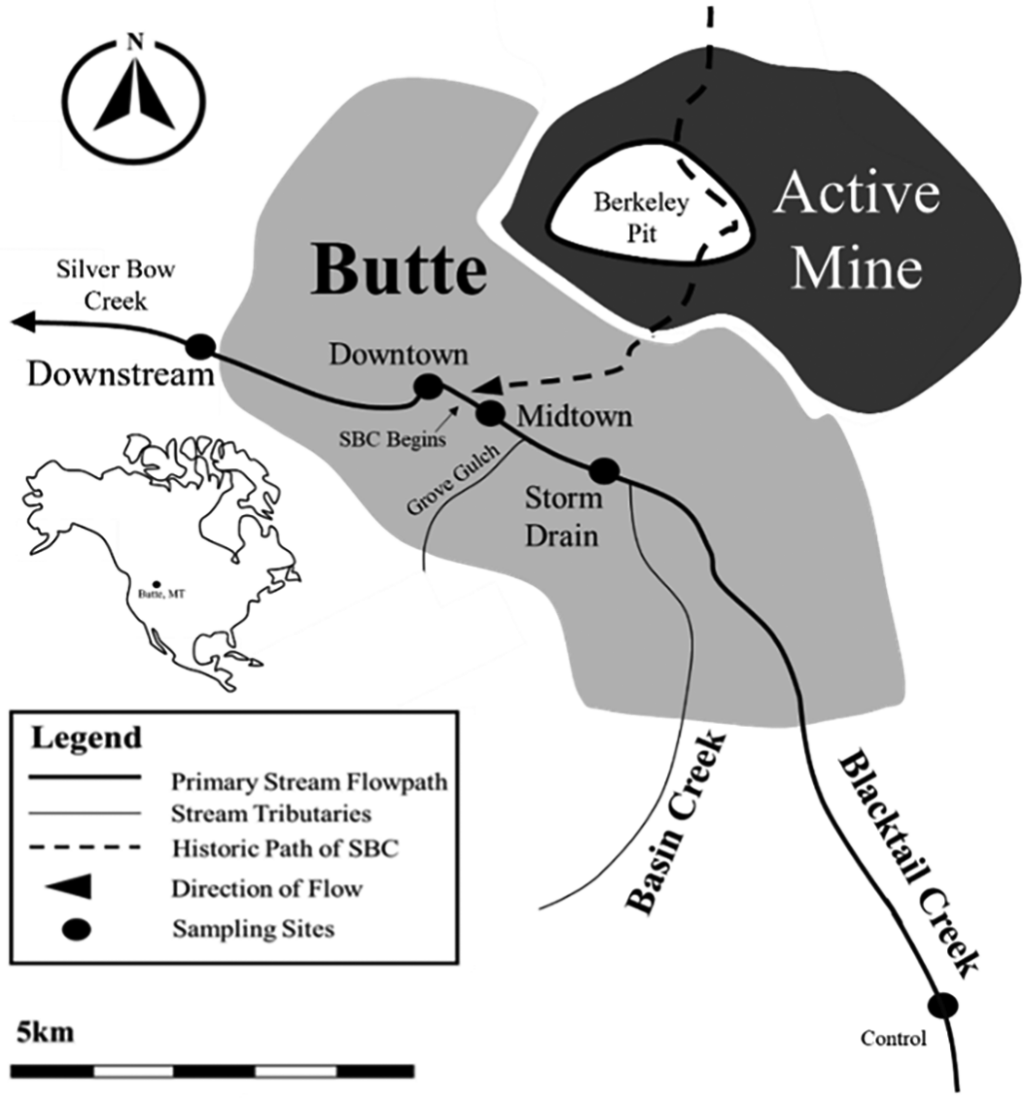
Silver Bow Creek, Blacktail Creek, and surrounding tributaries near Butte, MT. Black circles represent sites where concurrent surface water and metagenomic samples were collected. The insert map shows the study location in North America.

We selected five sampling sites to bracket the contamination epicentre (Figure 1). The upstream site on Blacktail Creek has been minimally affected by anthropogenic activity and served as the study's control site. All other sites were strategically placed in and around Butte near USGS monitoring stations (Figure 1). The Control and Midtown sites are located on Blacktail Creek. Sampling was conducted every 3 months from November 2015 to August 2016 to capture a temporal gradient across seasons. The Downtown and Downstream sites are located on Silver Bow Creek. The Storm Drain site is a small stormwater input to Blacktail Creek All sites are hereafter referred to as being located on SBC/BC (Silver Bow Creek/Blacktail Creek). Sixteen integrated metagenomic and geochemical samples were collected. Four samples each were collected from the Midtown, Downtown, and Downstream sites in November 2015, February 2016, May 2016, and August 2016. Two samples each were collected from Storm Drain and Control. Samples were collected from Control in May and November 2016. Storm Drain was sampled during November 2015 and May 2016 when stormwater actively flowed into SBC/BC. No stormwater was present at the site during the other sampling efforts.

The physiochemical conditions of the stream and the associated measurement errors were quantified in situ using handheld meters as previously described (Dahlquist Selking et al., 2023). Briefly, a WTW 3110 pH meter, YSI 30 conductivity meter, and DS5 Hydrometer were placed into the stream flow and measurements were recorded. Manufacturer errors are ±0.01 pH and ±0.5% for conductivity. A DS5 Hydrometer was used to measure DO and temperature. The DS5 Hydrometer has a manufacturer error of 0.5% and a detection limit of 5 μmol/L (Schmidt, 2017). Measurement errors are reported as five times the manufacturer error (±0.05 pH, ±2.5% DO, ±2% conductivity) or the field fluctuation error, whichever was higher (Data S1, Dahlquist Selking et al., 2023).

Water samples were collected for field spectrophotometry and fluid geochemical characterisation as previously described (Dahlquist Selking et al., 2023). Briefly, water samples were collected from the stream using high‐density polyethylene (HDPE) sampling scoop and divided into subsamples for field spectrophotometry and water filtration. Dissolved silica concentrations were determined using the Silicomolybdate Method 8185 (Hach test #656) and a Hach DR/2010 spectrophotometer. Water samples and field blanks were filtered into the prepared vials and stored until laboratory analysis. Briefly, each sample or blank was filtered to 0.2 μm using a 1.2 and a 0.2 μm 32 mm diameter (PALL® Acrodisc® Supor® membrane, hydrophilic polyethersulfone) sterile syringe filters, a TM clean 140 mL polypropylene syringe, nylon stopcock, 1 L HDPE Nalgene bottle, and Tygon XL‐60 tubing.

### Dissolved carbon analysis

DIC and DOC concentrations were measured in triplicate using an Aurora 1030 W Total Carbon Analyser system and autosampler. Analytical errors for both DIC and DOC measurements are ±3%. Standard solutions were analysed at the beginning and end of each analytical run to monitor instrumental drift. Standards (1, 5, 10, 20, and 50 ppm) for DIC analysis were prepared from lithium carbonate (Mallinckrodt, Analytical Reagent Grade) and sodium bicarbonate (Fisher Scientific, Certified A.C.S.). Standards (1, 5, 10, and 20 ppm) for DOC analysis were prepared from potassium hydrogen phthalate (ACROS Organics, Analytical Reagent Grade) and sucrose (Fisher Scientific; Certified A.C.S.). A calibration curve was then created using the raw CO_2_ data as integrated areas of signal peaks and the concentrations of the standards. The calibration curve was then used to convert the total CO_2_ concentrations of the samples to parts per million (ppm). Values were converted from ppm to molality assuming a solution density of 1 g/cm^3^. Additional standards were included at the end of each analytical run to monitor instrumental drift.

DIC was measured by reacting 8 mL of sample with 1.5 mL of 5% phosphoric acid (Baker Analyzed™, A.C.S. Reagent) at 70°C for 90 s, converting the DIC to gaseous carbon dioxide. The CO_2_ was then measured using a non‐dispersive infrared detector (NDIR) and reported as total CO_2_. DOC analysis was conducted by reacting 8 mL of sample with 1.5 mL of 5% phosphoric acid (Baker Analyzed™, A.C.S. Reagent) at 70°C for 270 s to purge DIC from the sample as CO_2_. The solution was then sparged with high‐purity nitrogen gas for 120 s to remove the CO_2_. The DOC was then converted to CO_2_ by reacting 8 mL of sample with 1.5 mL of 10% sodium persulfate (Fisher Scientific; Certified A.C.S) at 92°C for 120 s and the CO_2_ quantified by the NDIR for 180 s. Instrumental error for DIC/DOC analysis was calculated as the standard deviation of each set of sample triplicates (Dahlquist, 2017; Feldman, 2019; Law, 2018; Robertson, 2019; Schmidt, 2017).

### Major cation, major anion, and trace element analysis

Major cation, major anion, and trace element analysis were carried out at the Montana Bureau of Mines and Geology as previously described (Dahlquist Selking et al., 2023). Major cations including Li^+^, Na^+^, K^+^, Mg^2+^, and Ca^2+^ were analysed using a Fisher Scientific iCAP 6300 Series inductively coupled plasma optical emission spectrometer (ICP‐OES) using EPA method 200.7. Major anions including F^−^, Cl^−^, Br^−^, ${\mathrm{SO}}_{4}^{2-}$, ${\mathrm{PO}}_{4}^{2-}$, ${\mathrm{NO}}_{2}^{-}$ and ${\mathrm{NO}}_{3}^{-}$ were analysed using a Metrohm 882 Compact Ion Chromatograph (IC) Plus using EPA method 300.1. Trace elements concentrations including Li, Be, B, Al, Ti, V, Cr, Mn, Fe, Co, Ni, Cu, Zn, Ga, Ge, As, Se, Rb, Sr, Zr, Nb, Mo, Pd, Ag, Cd, Sn, Sb, Cs, Ba, La, Ce, Pr, Nd, W, Tl, Pb, and U concentrations were determined using a Thermo Scientific iCAP Q ICP‐mass spectrometry (MS) system using EPA method 200.8. All ICP‐OES, IC, and ICP‐MS measurements were accompanied by quality control standards to monitor instrumental drift. The geochemical data, reported in ppm or parts per billion (ppb), were converted to molality assuming a solution density of 1 g/cm^3^ (Data S1).

### Geochemical speciation modelling

Aqueous species calculations were conducted using the WORM (Water Organic Rock Microbe) Portal (Boyer, 2021; Boyer et al., 2021, 2022). The WORM Portal serves as an interface to the EQ3/6 software package, which has been extensively used to perform geochemical speciation modelling with an appropriate internally consistent thermodynamic database (Aiuppa et al., 2005; Helgeson et al., 1978; Howells et al., 2022; Johnson et al., 1992; Leong et al., 2021; Leong & Shock, 2020; Shock, 2009; Shock et al., 1989, 1997; Shock & McKinnon, 1993; St. Clair, 2017; St. Clair et al., 2019; Sverjensky et al., 1997; Wolery, 1992; Wolery & Jarek, 2003). The WORM thermodynamic database was recalibrated using thermodynamic data for metal carbonate complexes (St. Clair et al., 2019). The custom WORM database is valid at temperatures from 0 to 350°C and up to 200 bars. DO measurements were used to calculate PO_2_, which served as the redox potential for the geochemical speciation model. Concentrations lower than the detection limit were reported as half of the detection limit. Field blanks were examined for possible contamination. Blank concentrations exceeding 2% of the total sample concentration were subtracted from the total concentration. Only Mg and Fe values were adjusted based on blank concentrations. Analytes more concentrated in one or more blanks than in the samples (Al and Zn) were excluded from the analysis. Blank contamination (Al, Mg, and Fe) fell dramatically after ultra‐pure water washing procedures began in February 2016. Zinc contamination was persistent across the study period, presumably due to the samples contacting the black rubber syringe plunger during filtration. Missing DIC measurements were substituted for the average of DIC measurements at the site during the study period. The quality of chemical speciation calculations was assessed using a calculated charge balance. The resulting dataset of aqueous species was used to examine differences in copper speciation.

### Collection and analysis of metagenomic data

Sediment samples dedicated for DNA analysis were collected concurrently with geochemical samples using an HDPE scoop, homogenised in a sterile specimen cup, partitioned into 2 mL autoclaved plastic tubes, frozen on dry ice in the field and stored at −80°C until DNA extraction. DNA was extracted from collected sediments using a modified freeze–thaw and phenol‐chloroform extraction procedure (Brazelton et al., 2006; Dahlquist Selking et al., 2023; Howells et al., 2022; Huber et al., 2002). Briefly, the sediments were mixed with DNA extraction buffer amended with 2% (w/v) polyvinylpyrrolidone (Sigma‐Aldrich, molecular biology grade), homogenised and the cells lysed using three successive freeze–thaw cycles. The sediment was then treated with a lysosome solution, proteinase K, and a 20% sodium dodecyl sulfate (SDS) solution. Genomic DNA was then purified using a phenol/chloroform/isoamyl alcohol (24:24:1) solution, precipitated, treated with SEWS‐M Wash Solution (MP Biomedicals), eluted, and cleaned using PowerClean Pro DNA Clean‐Up Kit to a final extraction volume of 70 μL. Universal bacterial primers (1100F and 1492R) were then used to amplify the 16S rRNA genes present in the extracted DNA confirming that polymerase chain reaction‐amplifiable DNA was present and then discarded (Boyd et al., 2010). The DNA from each sample was then sequenced at the University of Minnesota Genomics Center using the Illumina HiSeq 2500 platform with 2 × 125 bp sequences.

Metagenomes were processed and the copper resistome was examined using the KBASE bioinformatics software platform (Chivian et al., 2023). FASTQ read files were imported using the Import FASTQ/SRA File as Reads from Staging Area app v1.0.58. All raw read files contained ~6.47 × 10^7^ reads of ~7.67 × 10^9^ bases with ≥33 Phred quality scores. Illumina adaptors were removed using the Trim Reads with Trimmomatic app v0.36 to generate clean read libraries. Read quality was assessed Assess Read Quality with Fast QC app v0.11.9. Reads were then assembled using MEGAHIT v1.2.9 using the meta‐large parameter preset. The resulting metagenomic datasets were annotated and screened for genes related to copper resistance (the copper resistome) using the Annotate Metagenome Assembly and Re‐Annotate Metagenome with Rapid Annotation Using Subsystems Technology toolkit (RASTk) v1.073 genetic analysis program (Brettin et al., 2015; Keegan et al., 2016; Overbeek et al., 2014). RASTk classifications are based on the SEED database and indicate the number of genes in each metagenome with roles mapping to SEED subsystem classes and identifications. The specific functions of all copper resistance genes are not entirely understood. All functional designations were based on published experimental data, the Kyoto Encyclopedia of Genes and Genomes (KEGG) database, or the BacMet (Antibacterial Biocide & Metal Resistance Genes) (Kanehisa, 2019; Kanehisa et al., 2023; Kanehisa & Goto, 2002; Pal et al., 2014) Annotated genes belonging to the ‘Stress Response, Defense and Virulence’, ‘Copper Homeostasis: Copper Tolerance’, ‘Copper Transport and Blue Copper Proteins’ and ‘Copper Transport System’ SEED subsystem designations or annotations that are currently not assigned to a subsystem were individually examined to determine if they are involved in copper resistance (Cooksey, 1994; Dupont et al., 2011; Fong et al., 1995; Ladomersky & Petris, 2015; Latorre et al., 2011; López et al., 2018; Padilla‐Benavides et al., 2013; Raimunda et al., 2011; Vulpe, 1995). Other genes related to copper belonging to the ‘Respiration’, ‘Nitrogen Metabolism’ or ‘Cell Cycle, Cell Division and Death’ subsystems were discarded as these are not involved in reducing copper stress. Hypothetical and other predicted proteins that could not be traced to a specific function were discarded. Selected copper resistance genes were quantified using the gene counts generated by the MG‐RAST analysis and sorted into functional groups based on mode of action to form discrete metabolic profiles related to environmental copper resistance, the copper resistome. Predicted *CopC*, *CusS*, *CusR*, *CpxR*, *CpxA*, *ScsD*, *CutE*, *CutC*, *CopZ*, and periplasmic copper tolerance (PCTP) genes were designated as intercellular binding and signalling (ICBS) genes as they are involved in intercellular trafficking, sequestration, signalling, transcription regulation or other similar functions. Predicted *CutA*, *CopB*, *CorC*, *CusB*, *CusC*, *CusA,* and multi‐metal ATPase proteins were designated as efflux genes as they export copper and/or other cations across cellular membranes to reduce oxidative stress related to copper toxicity. Predicted multicopper oxidases were designated as transformation genes given their role in protecting periplasmic proteins from Cu(I) mediated toxicity (Dupont et al., 2011; Ladomersky & Petris, 2015). Predicted *CopD* genes were designated as uptake genes as *CopD* is an inner membrane protein involved in copper uptake that helps regulate intercellular copper concentrations (Cooksey, 1994). An unnamed membrane copper tolerance protein (HMAND) was designated as other. Similarities in resistomes were examined using diverging heatmaps. Hierarchical clustering of resistomes by site and month was performed in PAST statistical software using Ward's method (Hammer et al., 2005).

### Integrated metagenomic and geochemical statistical modelling

The geochemical data matrix used for geochemical speciation modelling of 36 geochemical variables was paired with the associated copper resistome to form an integrated dataset suitable for ordination. Be, Co, Zr, Nb, Pd, Ag, Cd, Sn, Cs, La, Ce, Pr, Nd, Pb, Tl, and Th were undetected in all samples and excluded from further analysis. The geochemical data were scaled using Z‐conversion (Nishiyama et al., 2018). Geochemical contributions to variation in the resistomes were assessed using redundancy analysis (RDA) in PAST statistical software to identify primary geochemical contributors to variation in the metagenomes (Hammer et al., 2005; Nishiyama et al., 2018; Weeks et al., 2023). Variables with large loadings (±0.30) were selected for further analysis (Weeks et al., 2023). All other geochemical variables were discarded. The dissimilarities in copper resistome composition were assessed using principal coordinates analysis (PCoA) calculated using the Jaccard dissimilarity index. The biplot was used to examine the spread of observations along the two dimensions that account for the most variability in the dissimilarity matrix. The ‘envfit’ function (with 999 permutations) of the ‘vegan’ package for R v3.4.2 was used to assess the correlations between the metagenomic ordination and the selected environmental parameters (*α* = 0.05) (Dixon, 2003; Weeks et al., 2023).

## RESULTS

### Aqueous geochemistry

SBC/BC exhibited dynamic temperatures, ranging from 3.2 ± 0.5°C during November 2015 to as high as 17.7 ± 0.5°C in August 2016. The stream maintained a circumneutral pH ($\bar{x}$_*n*=16_ = 7.76 ± 0.05), with samples from the Storm Drain site representing both the lowest (pH 7.07 ± 0.05) and highest (pH 8.41 ± 0.05) extremes during the study period. All samples exhibited high DO concentrations ($\bar{x}$_*n*=16_ = ~3 × 10^−4^ mol/kg, ~9.6 ppm). Conductivity varied between samples, ranging from 144 ± 3 to 572 ± 11 μS/cm. DOC concentrations ranged from 1.9 ± 0.1 to 8.2 ± 0.2 ppm (Data S1).

**FIGURE 2:**
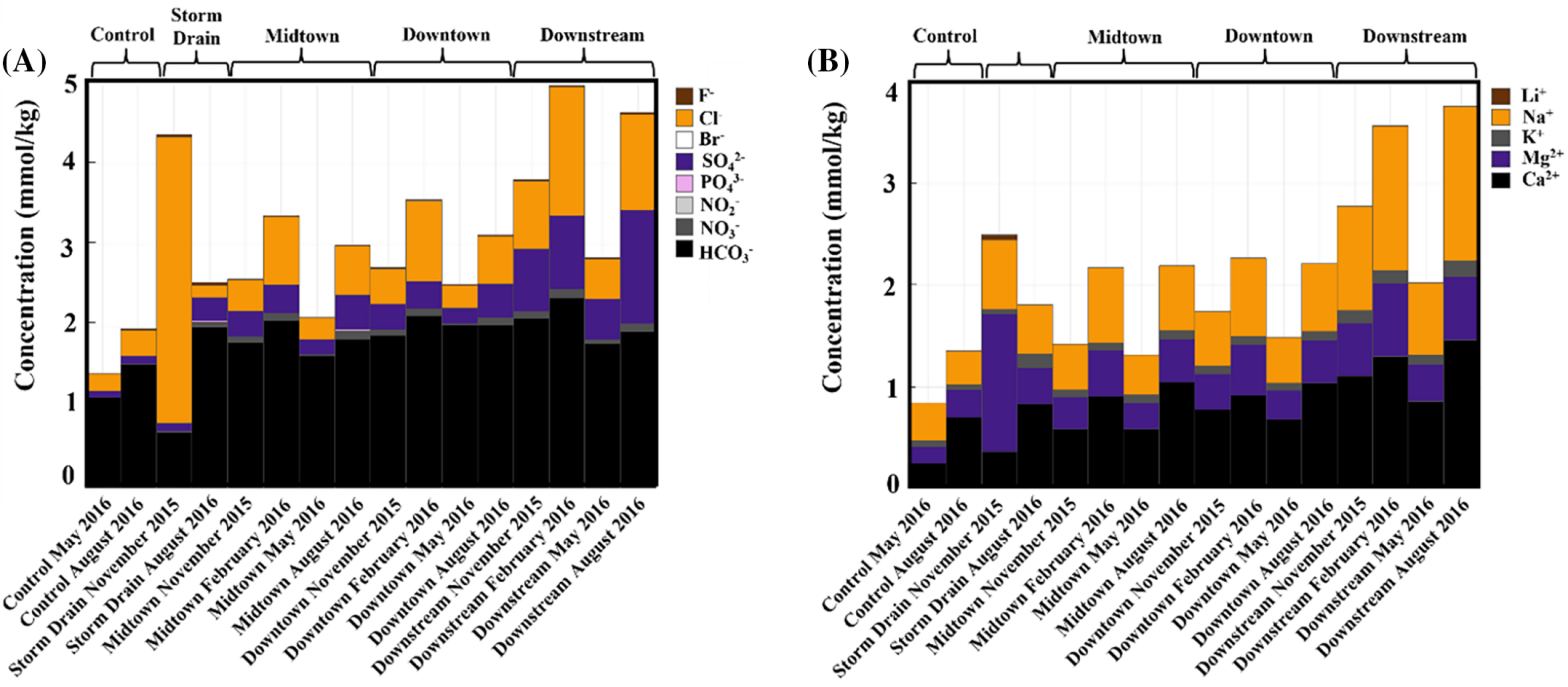
Major (A) anion and (B) cation concentrations in Silver Bow Creek and Blacktail Creek.

The Control, Midtown, Downtown, and Downstream sites exhibited similar major cation and anion profiles (Figure 2). The Control site exhibited the lowest dissolved major anion and cation concentrations, whereas the Downstream site exhibited the highest concentrations. Bicarbonate, chloride, and sulfate dominated the Control, Midtown, Downtown, and Downstream anion profiles. Anion profiles primarily varied in chloride and sulfate concentrations, whereas bicarbonate and nitrate concentrations remained similar at all sites. Calcium concentrations generally increased downstream, whereas sodium and magnesium concentrations varied substantially by site and season. The Storm Drain in November 2015 exhibited ~4.4 × $\bar{\mathrm{Cl}}$ and ~2.9 × $\bar{\mathrm{Mg}}$ concentrations (Figure 2A, Data S1). Calcium, sodium and magnesium dominated the cation profiles at all sites (Figure 2B).

**FIGURE 3:**
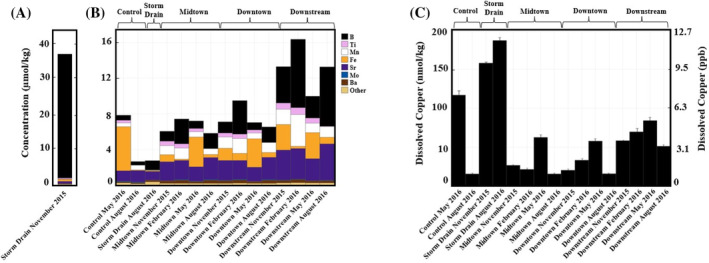
Trace element and copper concentrations in Silver Bow Creek and Blacktail Creek. Elements representing less than 1% of the total trace element composition, including V, Cr, Cu, Ga, As, Se, Rb, Mo, Sb, W, and U, are pooled and designated as ‘other’. (A) Trace element concentrations in Storm Drain November 2015. Note the differing scales. (B) Trace element concentrations arranged by site according to site by time. (C) Total dissolved copper concentrations in Silver Bow Creek and Blacktail Creek. Error bars represent ICP‐MS measurement errors.

Trace element composition varied substantially between sites (Figure 3, Data S1). The Storm Drain in November 2015 exhibited ~7.8 × $\bar{\text{Boron}}$ (Figure 3A, Data S1). Boron, iron and strontium made up the largest proportions of all trace element assemblages, whereas other trace elements varied (Figure 3B). Strontium concentrations rose at the Midtown, Downtown, and Downstream sites. Iron concentrations varied substantially across sites and seasons. Metal(loid) concentrations, notably boron, spiked at the Downstream site. Other trace element concentrations are included in Data S1.

**FIGURE 4:**
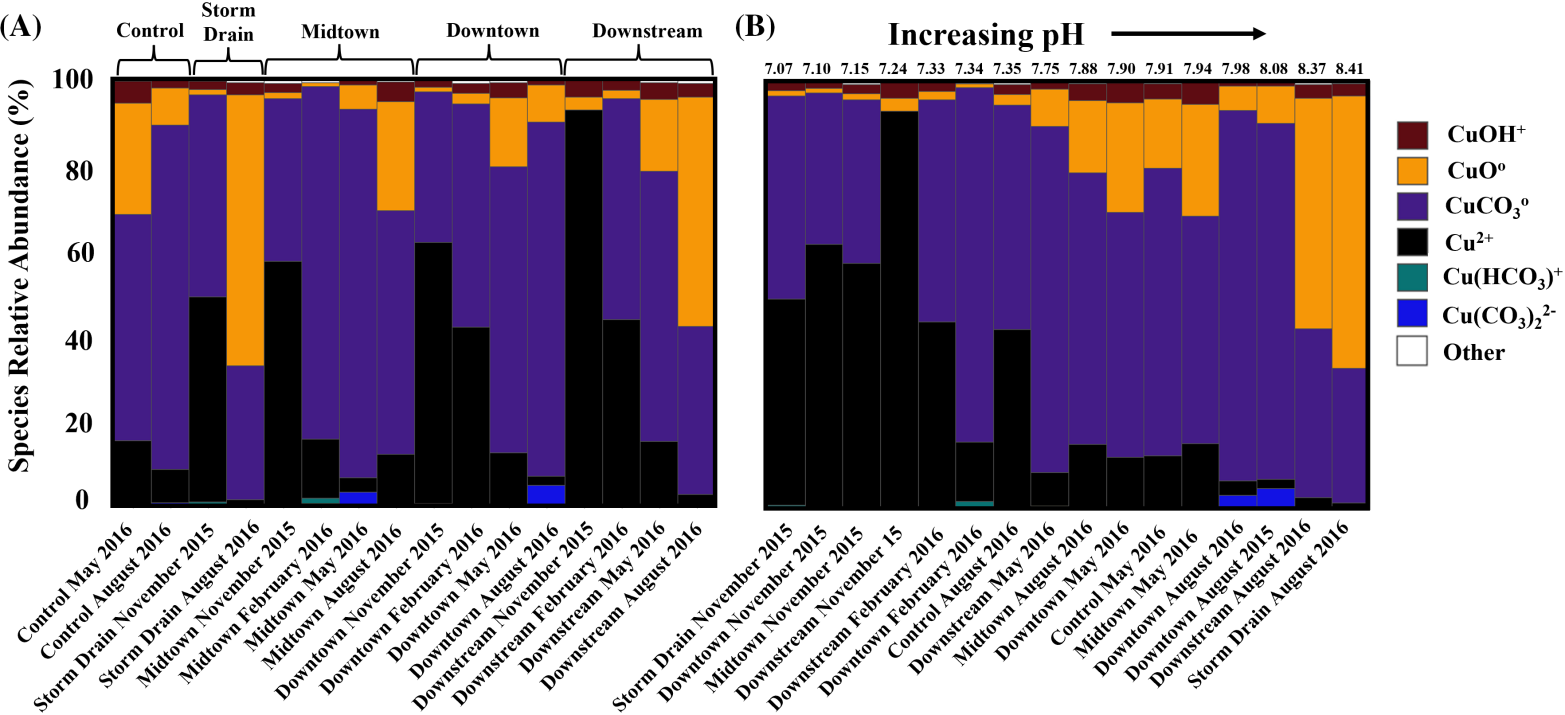
Copper species relative abundances in Silver Bow Creek and Blacktail Creek. Relative abundances are calculated using the mass contribution of each species to the total dissolved copper concentration (Figure 3C). Copper speciation profiles are arranged according to the site, (A) by time and (B) pH.

SBC/BC contained low, highly variable copper concentrations ($\bar{x}$_*n*=16_ = 62 nmol/kg, standard deviation = 51 nmol/kg) (Figure 3, Data S1). Three samples exhibited unusually high copper concentrations relative to the other samples. The Storm Drain contained 157 ± 2 nmol/kg (~2.5 × $\bar{\mathrm{Cu}}$) November 2015 and 187 ± 5 nmol/kg (~3 × $\bar{\mathrm{Cu}}$). The Control in May 2016 contained 117 ± 2 nmol/kg (~1.8 × $\bar{\mathrm{Cu}}$). No other temporospatial copper concentration trends occurred. Geochemical speciation modelling of all samples produced quality speciation calculations (Data S2). All samples fell well within the ±10% charge balance error limit ($\bar{x}$_*n*=16_ = −0.42% charge imbalance eq/kg H_2_O). Total charge balance ranged from +7.02% to −8.72% with 13 of 16 charge balance errors falling within ±5% (Data S2). The Control site exhibited both the largest positive (7.02%) and negative (−8.72%) charge balance errors. All observations from February 2016 fell within ±1% charge balance error. Copper is primarily speciated as Cu^2+^, ${\text{CuCO}}_{3}^{o}$ and CuO^o^. $\mathrm{Cu}{\left({\mathrm{CO}}_{3}\right)}_{2}^{2-}$, Cu(HCO_3_)^+^ and CuOH^+^ accounted for small proportions of the total copper species assemblages. Cu^2+^ dominated at lower pH (7.05–7.35 ± 0.05) and peaked during November before falling in abundance in May and August (Figure 4A). ${\text{CuCO}}_{3}^{o}$ displayed an inverse relationship with Cu^2+^ abundance. CuO^o^ displayed similar seasonal trends with ${\text{CuCO}}_{3}^{o}$, presumably driven primarily by rising pH (8.37–8.41 ± 0.05) in August 2016 (Figure 4B, Data S3).

### Multicopper oxidases dominated metagenomes

**FIGURE 5:**
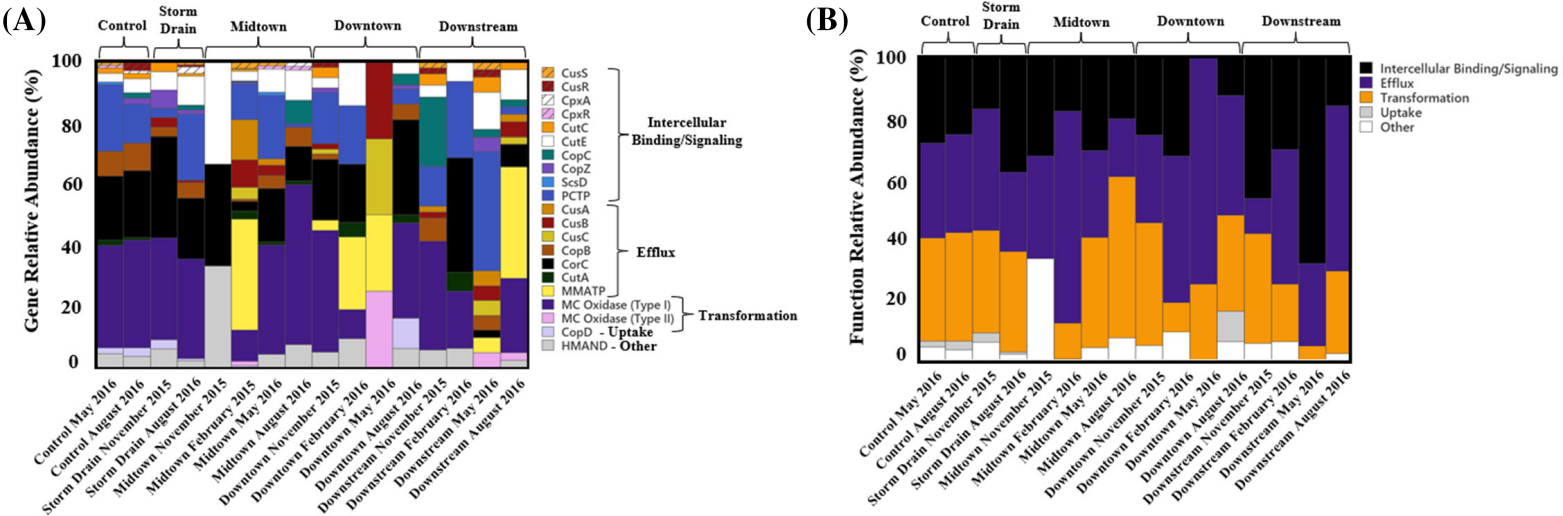
Silver Bow Creek and Blacktail Creek microbial copper resistance (A) gene and (B) functional relative abundances.

RASTk analysis detected 21 copper resistance genes in the 16 metagenomes (Data S4). Multicopper oxidase genes (Type I) comprised ~26% of all annotated copper resistance genes (Figure 5A). Multimetal ATPases, Mg/Co efflux genes (*CorC*), and PCTP genes comprised smaller proportions of the total resistome, accounting for ~12%, ~14%, and ~14%, respectively. All other genes accounted for <12% of the resistome (Figure 5A). All resistomes varied substantially in complexity and composition. Some metagenomes contained as few as three copper resistance genes, although most communities exhibited 10+ copper resistance mechanisms.

Intracellular binding and signalling genes dominated all resistomes (Figure 5B). Efflux and transformation genes made up similar abundances of copper resistomes. Transformation gene relative abundance generally peaked in February and was especially high in May 2016 at the Midtown site. Efflux genes spiked at the Downstream site in May 2016. Uptake genes made up low abundances of all resistomes.

### Integrated geochemical–metagenomic statistical modelling suggested geochemistry correlated with the copper resistome

**FIGURE 6:**
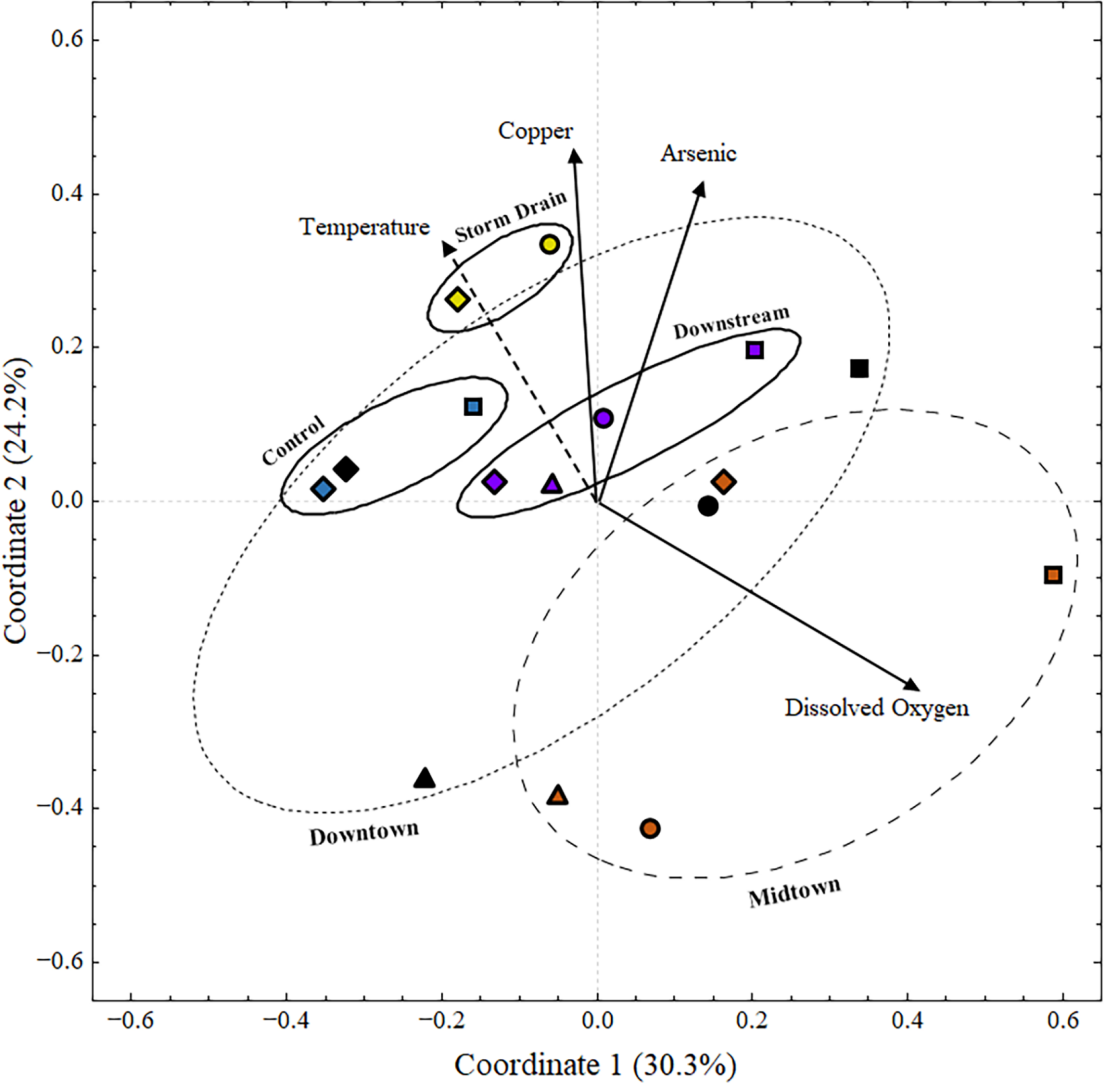
Principal coordinates analysis ordination calculated using the Jaccard dissimilarity matrix of the occurrence of copper‐related genes in 16 metagenomes from Silver Bow Creek and Blacktail Creek. Significantly correlated geochemical parameters are fit to the ordination using the ‘envfit’ function in R. Points represent the copper resistome of each discrete metagenome. Vectors represent geochemical variables. Significantly correlated (*p* ≤0.05) parameters are represented with a black line. Parameters that approach significance (*p* ≤0.1) are represented using a dotted line. Colours indicate a site (blue = Control, yellow = Storm Drain, orange = Midtown, black = Downtown, purple = Downstream). Shapes indicate season (triangle = February, square = May, diamond = August, circle = November). Closely related resistomes (Control, Storm Drain, Downstream) from the same site are grouped using black circles. More variable resistomes from the same site are grouped using dashed circles (Downtown, Midtown).

Silver Bow Creek and Blacktail Creek copper resistome composition correlated with the creek's geochemical profile (Figure 6). The first three components accounted for approximately 67% of the total variance within the dataset, suggesting PCoA contained useful information on the relationships between the 36 environmental parameters and 21 copper resistance genes. Variation in each microbial community's resistomes appeared to be related to spatial differences as sample location drove variance in the resistomes. The metagenomes at the heart of Butte (Midtown and Downtown) exhibited highly variable resistomes compared to those collected at the Storm Drain and the Control site (Figure 6). RDA suggested that seven parameters (temperature, DO, copper, arsenic, phosphate, manganese, and titanium) contributed to microbial resistome variation. Fitting the environmental parameters to the ordination indicated that three (copper [*p =* 0.024], arsenic [*p =* 0.034], and oxygen [*p =* 0.022]) of seven water quality parameters significantly correlated with changes to the microbial copper resistomes. Temperature (*p =* 0.087) approached significance. Phosphate, manganese, and titanium were not significantly correlated and excluded from further analysis. Temperature and DO exhibited an inverse relationship, whereas copper, arsenic, and temperature correlated with one another (Figure 6).

## DISCUSSION

As the world has transitioned into the digital age, humanity has become more reliant on copper resources. The increasing population, industrialisation, and economic expansion all increase the global demand for copper‐intensive products (Carvalho, 2017; Mudd, 2007). As life cycle assessments (LCA) predict that the environmental impacts of copper mining and production will more than double by 2050 compared to 2010, streams worldwide are in danger of mining‐related degradation (Dong et al., 2019; Jiao et al., 2023; Moore & Langner, 2012; Moore & Luoma, 1990; Owens et al., 2023). Ecological insight into impacted areas is paramount to protecting and restoring vulnerable aquatic ecosystems (Di Toro et al., 2001; Cavicchioli et al., 2019).

As previously discussed, other environmental microbial surveys investigate parameter‐specific geochemical analysis, but microbial‐geochemical integration needs further innovation (Li, Hu, et al., 2021). Most lotic microbial ecology survey studies focus solely on metagenomic analysis, analyse metal concentrations without statistical integration, examine metal concentrations without full geochemical context, consider only a reduced geochemical subset, do not consider speciation, or analyse metal enrichment relative to human health standards (Li, Chen, et al., 2021).

Here we present a statistical framework that integrates a fully realised geochemical context with metabolic insight into complex microbial communities. Our analysis of 36 environmental parameters related to 21 copper resistance genes in 16 metagenomes indicates microbial copper resistome structure is correlated with copper (*p =* 0.024), arsenic (*p =* 0.034) and DO (*p =* 0.022) (Figure 6). Here, we discuss relationships between copper and arsenic, the difference between resistomes, seasonal connections between copper speciation and resistome variation, possible connections between groundwater and microbial ecology, and metal ecotoxicology in mining alter ecosystems. Connections between microbe functional potential and geochemistry suggest that copper stress is likely controlled by cold weather‐modulated speciation changes and additive interactions with arsenic. Copper stress is linked to the more anthropogenically impacted areas and may be used to trace contamination influx.

### Seasonal sublethal copper and arsenic exposure altered local resistomes

Geochemistry altered microbial copper resistance metabolic profiles through multiple co‐occurring metal(loid) stressors (Figure 6). Although we initially anticipated that the relatively low copper concentrations observed in this study might not produce traceable signatures in the metagenomes, even low ($\bar{x}$_*n*=16_ = 62 nmol/kg) copper levels appear to be relevant to microbial ecology (Albright & Wilson, 1974; Flemming & Trevors, 1989). These findings are consistent with multiple other studies of sublethal copper exposure to microbes and suggest that even lower copper concentrations than previously analysed (110.14 nmol/kg) depress microbial activity (Albright & Wilson, 1974; Domek et al., 1984; Nielsen & Wium‐Andersen, 1970).

Connections between arsenic, copper, and resistomes suggest that the metal(loids) may have additive effects on microbial stress. Previous arsenic toxicity studies using automated ribosomal intergenic spacer analysis (ARISIA) support additive stress effects as arsenic‐copper exposure had unusual effects on microbial sediment communities (Mahamoud Ahmed et al., 2018). Exposure to arsenic alone had minimal impacts on the genetic structure and functional profile, whereas copper exposure severely depressed microbial activity. Exposure to both copper and arsenic simultaneously created a separate and pronounced effect on the microbial communities compared to copper alone. Invertebrate culture experiments examining copper and arsenic effects on *Daphnia magna* support metal(loid) co‐exposure in initiating population‐wide changes (Asselman et al., 2019). The co‐exposed *Daphnia* did not exhibit straightforward relationships between genetic variation and metal stress. Instead, co‐exposed populations exhibited genome‐wide transcriptomic changes and altered moulting dynamics at the population level. Vertebrate studies further underline arsenic and copper‐induced oxidative stress cascades and inflammation not observed under single metal(loid) exposure (Shao et al., 2018; Wang et al., 2017). Other phytotoxicity studies suggested that additive bioavailability models cannot adequately predict arsenic and copper co‐exposure impacts on plants (Kader et al., 2018). Although more research is needed to fully explore the metal(loid) mixtures impacts on microorganisms, our results add to the growing evidence that concurrent arsenic and copper exposure is ecotoxicologically relevant.

Beyond metal(loid) exposure, seasonal variation controlled shifting copper resistomes (Figure 6). Although DO dramatically alters metal speciation and bioavailability, changing DO levels presumably did not greatly alter metal bioavailability given similarly high DO concentrations at all sites ($\bar{x}$_*n*=16_ = ~3 × 10^−4^ mol/kg, ~9.6 ppm) (Feldman, 2019). If oxygen‐modulated speciation changes are relevant to microbial copper resistance, it is possible that even subtle speciation shifts altered metal bioavailability enough to elicit a metabolic response. As temperature approached significance, however, we assume oxygen did not drive metal bioavailability. DO variation in freshwater is primarily controlled by respiration and photosynthesis rates and temperature‐mediated oxygen solubility at high latitudes (Petsch, 2003). As the DO grab samples used here are midday observations and do not measure river metabolism, temperature variation likely drove resistance profile changes (Figures 6 and 8).

**FIGURE 7:**
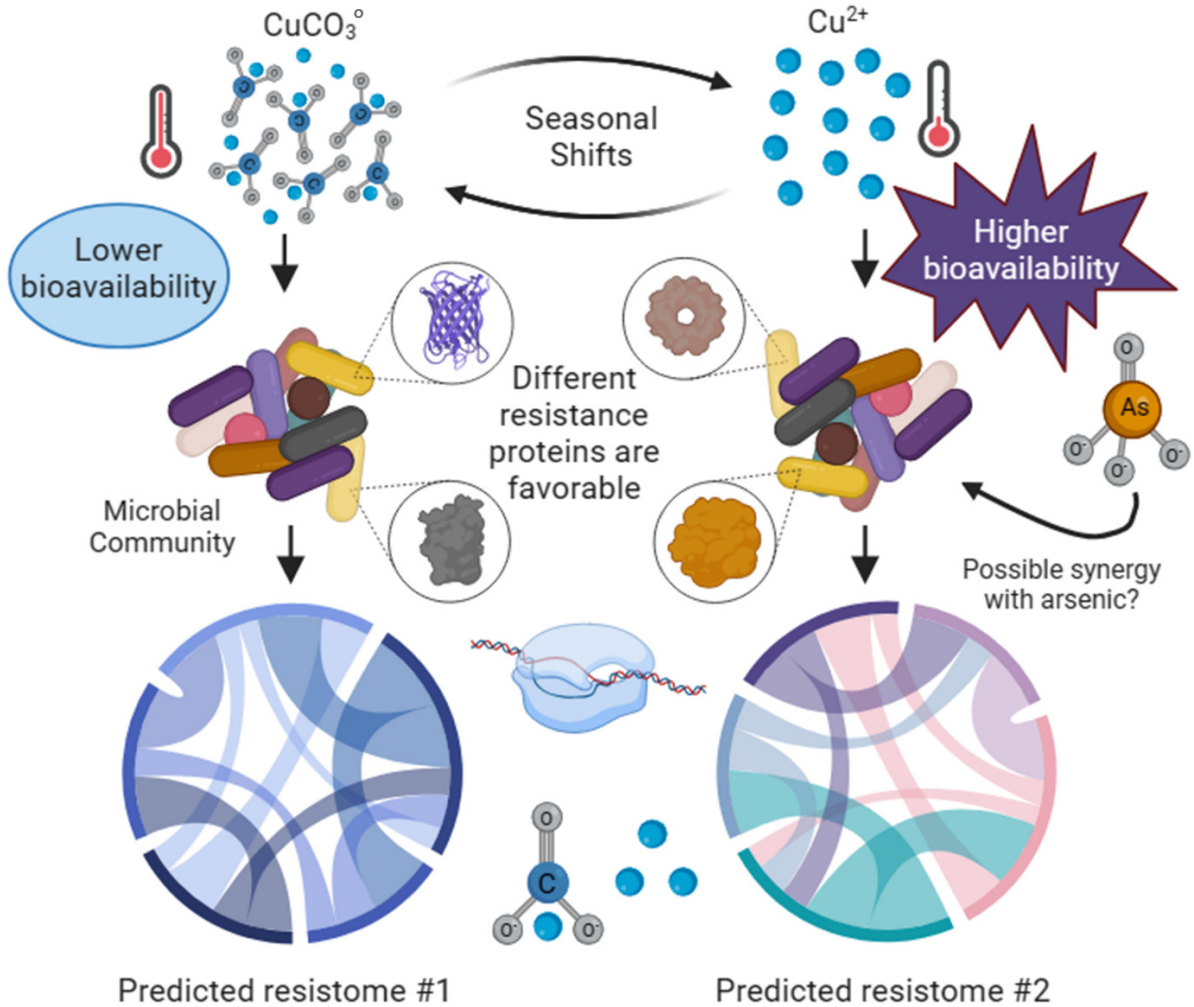
The predicted relationship between Silver Bow Creek and Blacktail Creek copper seasonality, copper bioavailability and microbial copper resistance. Seasonal shifts in Cu^2+^, ${\text{CuCO}}_{3}^{o}$ and arsenic exposure alter local microbial copper resistome composition. Predicted resistomes are visualised as intertwined nodes to represent metabolic pathways.

If a seasonal variation is indeed relevant to microbial copper resistance, then presumably seasonally linked arsenic or copper stress changed the copper resistomes. Although neither parameter exhibited a strong concentration temporal trend, copper speciation varied substantially between seasons (Figure 3C, Data S1–S3). Indeed, seasonally linked copper speciation supports connections between seasonal shifts and changes in copper bioavailability, that is, increased abundances of the cupric ion in November (Figure 4). As such, we propose that seasonal copper speciation changes are the primary driver altering copper resistome structure with arsenic as a secondary contributor (Figure 7).

### Resistomes exhibited temporally and spatially distinct patterns

**FIGURE 8:**
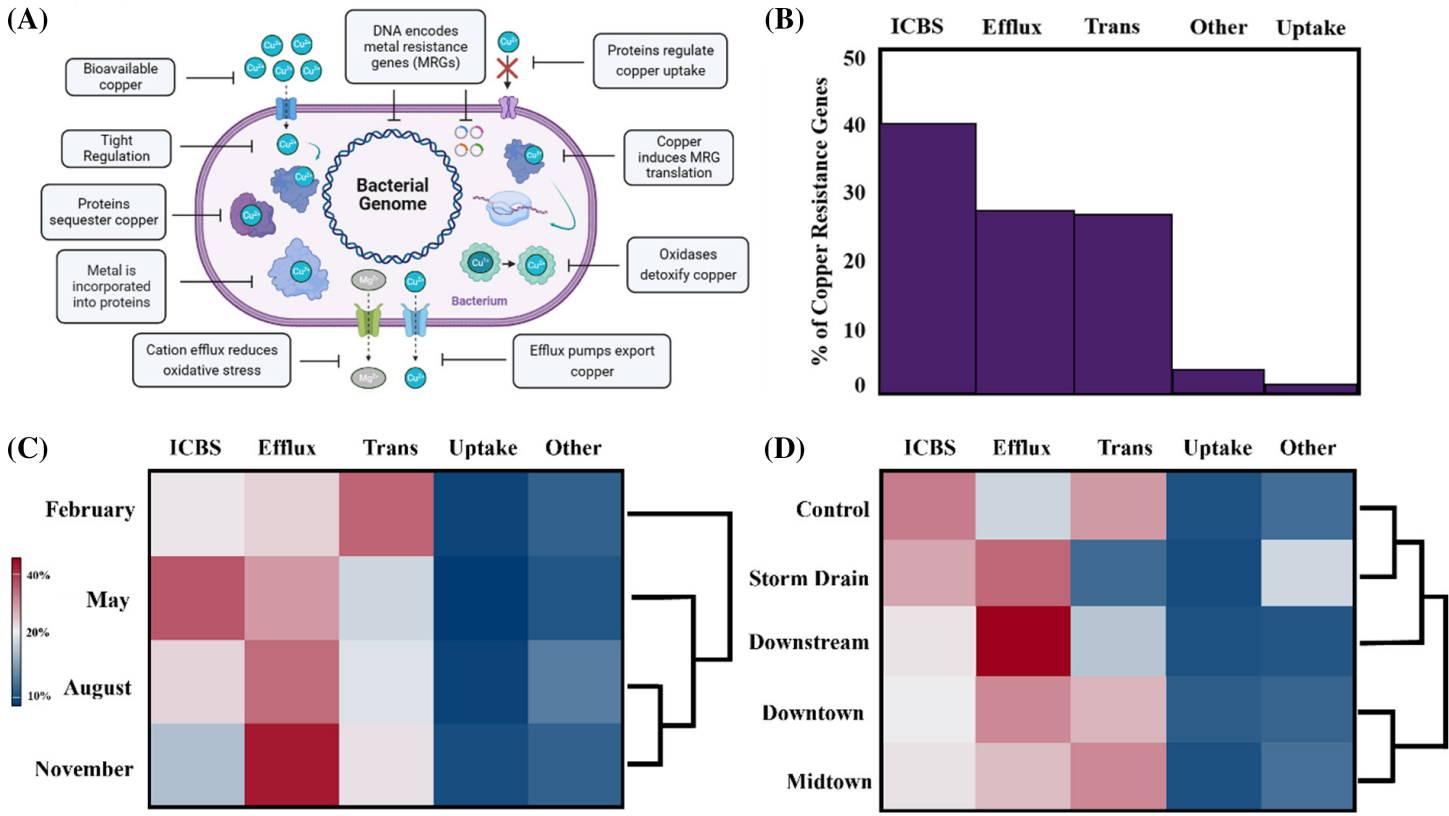
(A) Copper resistance gene functional groups used by bacteria to reduce copper stress. (B) Copper resistance gene functional category relative abundances in all metagenomes (Efflux, cation efflux; ICBS, intercellular binding and signalling; Trans, transformation). (C) Copper resistance gene functional category relative abundances arranged by month. (D) Copper resistance gene functional category relative abundances arranged by site. Red colours indicate high abundances (>20% of the resistome), white colours indicate medium abundances (~20% of the resistome) and blue colours indicate low abundances (<20%) of the resistome. The colour strength is proportional to higher divergence from the mean. Hierarchical clustering dendrogram linkages are calculated using Ward's method and represent similarities between the resistomes.

Microbes in Silver Bow Creek and Blacktail Creek exhibited complex cellular machinery to reduce copper stress (Figure 8A). Gene relative abundances in the five primary functional categories fluctuated considerably, although ICBS genes dominated all resistomes (Figure 8B). Efflux and transformation genes had similar overall abundances but differed between months. Although we initially expected similarities in November and February resistomes due to cold temperatures and low flow regimes, copper efflux genes relative abundance peaked in November before transformation gene relative abundance peaked during February (Figure 8C,D). Microbes in SBC/BC appeared to rely on both intercellular detoxification and efflux to respond to copper exposure (Figure 8B). The detoxification, efflux, and sequestration resistance mechanisms observed in conjunction with numerous genes involved in intercellular copper trafficking indicate that molecular responses to copper are multifaceted, with chaperone proteins regulating cellular stress responses (Figure 8A).

### Altered resistomes may be linked to groundwater

Similarities between microbial resistomes at Downtown and Midtown suggest that altered resistomes may be linked to groundwater inputs near Butte (Figures 6 and 8D). As previously discussed, other studies suggest that contamination enters the stream as groundwater inputs (Lund, 2018; Radar, 2019; Robertson, 2019; Tucci & Icopini, 2012). There is some evidence that groundwater inputs cause unusual microbial activity in Butte, especially Downtown. Experiments at Downtown revealed sulfate reducing bacteria in the water column. Oxic surface water generally does not support sulfate reduction, suggesting that the microbes entered the stream from the sub‐oxic groundwater below (Robertson, 2019). Differences in sediment pore water chemical gradients support groundwater entering the creek at the site as sulfate increases with depth at the site (Radar, 2019). If groundwater entered the stream in Butte, it may explain the variability in the Midtown and Downtown resistomes. Groundwater could have altered the stream's geochemistry or carried foreign microbes. Groundwater altering the microbial resistomes is further supported by the lack of variability in Control, Storm Drain, and Downstream resistomes, presumably due to them not receiving groundwater inputs (Figure 8D).

### Improving integrated microbe‐metal stress models

The trends in both geochemistry and microbial metabolic functionality underline the relationship between microbial copper resistance and environmental conditions. Further sampling and metagenomic statistical modelling should be conducted to improve the integrated methods presented in this study and fully explore the geochemical effects on microbial metabolic responses. Adding these data to other geochemical‐metagenomic datasets will strengthen biogeochemical insights. Future research should focus on identifying which microbes are harbouring metal resistance genes and linking their abundance to geochemical changes. Given the highly specific resistome signatures featured in this study, microbial assemblages utilising copper resistance genes are likely equally dynamic.

It is worth noting that RDA analysis suggested that several other geochemical factors (phosphate, manganese, and titanium) contribute to variation in copper resistomes, although their contribution to resistome structure was not significant. Their contribution to the RDA could indicate that other geochemical factors have effects on copper resistomes and could be investigated further. Moreover, some geochemical factors related to microbial metal resistance are partially controlled by microbial metabolism. It is possible that DO changes implicated in this study are actually a result of microbial metabolic responses to metal stress. Finally, although sediment microbiological analysis and surface water geochemistry are useful in understanding the creek microbial communities, sediment pore water analysis may contribute depth to future studies given the differences between pore water and the overlaying surface water (Radar, 2019). Copper, arsenic, and DO pore water characterisation could clarify interactions between microbial metabolism and geochemistry.

## CONCLUSIONS

Contaminant geochemistry shapes metal bioavailability and alters microbial metal stress responses. Even sublethal conditions appear to drive changes in metabolic potential, presumably due to certain microbes being better equipped to cope with changing metalloid coexposure. Further exploration of the relationship between resistomes and local geochemistry beyond these 16 metagenomes and 36 geochemical parameters could be useful in determining the extent of anthropogenic degradation and how to mitigate it. Future resistome characterisation could investigate microbial community arsenic resistomes to help clarify connections between copper and arsenic. Correlations among multiple resistomes and geochemical context could be useful in examining connections between microbial metabolic signatures and metalloid mixtures. Further methodological innovation, complete geochemical profiles and standard methods will strengthen future microbial insights. Although our results are inherently constrained by sample size and may not drastically shape environmental policy, this analysis is the first step in our ongoing initiative to help environmental managers make biogeochemically and ecotoxicologically informed decisions.

As mining is a vital part of humanity's technologically complex and equitable future, we hope this study can help minimize the unintended consequences of necessary resource extraction. We are just beginning to understand how microbial ecology can be used to mitigate anthropogenic degradation. The methods presented in this study are ultimately a theoretical framework that can be used in other systems to easily examine how habitat geochemistry shapes the foundational communities that support higher trophic levels (fish, macroinvertebrates, birds) typically targeted during ecological restoration. Our methods could easily be adapted to examine other relevant microbial processes such as carbon or nitrogen cycling. Moreover, we posit that Silver Bow Creek and Blacktail Creek are useful experimental settings for investigating microbial ecology, geochemistry, and biogeochemical interactions. Understanding metal biogeochemical processes in the area is critical for creating effective management solutions. The anthropogenic impacts at the stream's headwaters and the Clark Fork River below make SBC/BC a model ecosystem that will yield useful insights into mining‐impacted rivers worldwide. Similar biogeochemical studies can help bridge the gap between water quality and ecological functionality, identify restoration targets for action, provide insight into microbial activity, and establish genetic targets for bioremediation.

## AUTHOR CONTRIBUTIONS

**Paul G Helfrich:** Conceptualization; methodology; data curation; validation; formal analysis; investigation; visualization; writing – original draft; writing – review and editing. **Johnathan Feldman:** Methodology; data curation; validation; formal analysis; investigation; visualization; writing – review and editing. **Eva Andrade‐Barahona:** Methodology; validation; investigation; writing – original draft; writing – review and editing. **Isaiah Robertson:** Methodology; data curation; validation; investigation; writing ‐ review and editing. **Jordan Foster:** Methodology; data curation; validation; investigation; writing – review and editing. **Renee Hofacker:** Methodology; data curation; validation; investigation; writing – review and editing. **Gavin Dahlquist Selking:** Methodology; data curation; validation; investigation; writing – review and editing. **Cody S Sheik:** Methodology; data curation; validation; investigation; resources; writing – review and editing. **Alysia Cox:** Conceptualization; methodology; data curation; validation; formal analysis; supervision; funding acquisition; investigation; visualization; project administration; resources; writing – review and editing; writing – original draft.

## CONFLICT OF INTEREST STATEMENT

The authors declare no conflicts of interest.

## Supporting information


**Data S1.** The complete geochemical profiles associated with each metagenome were analysed in this study.
**Data S2.** The complete set of geochemical speciation profiles generated by the Water Rock Organic Microbes portal (WORM Portal) analysis software.
**Data S3.** The relative abundance of aqueous copper and arsenic species was calculated using Water Rock Organic Microbes portal (WORM Portal) analysis software.
**Data S4.** The copper resistance gene counts (the copper resistome) indicate the number of genes mapping to each resistance function in the SEED genomic database.

## Data Availability

All geochemicalparameters, calculated speciations, and copper resistance gene counts are included in the supplementary information and available on Zenodo as an excel file (Helfrich et al., 2024). Metagenomic sequence read data are available in the NCBI database under the project accession PRJNA1186244.
